# Efficacy and safety of canagliflozin in subjects with type 2 diabetes and chronic kidney disease

**DOI:** 10.1111/dom.12090

**Published:** 2013-03-28

**Authors:** J-F Yale, G Bakris, B Cariou, D Yue, E David-Neto, L Xi, K Figueroa, E Wajs, K Usiskin, G Meininger

**Affiliations:** 1 Department of Medicine, Royal Victoria Hospital and McGill UniversityMontreal, Canada; 2 Department of Medicine, The University of Chicago MedicineChicago, IL, USA; 3 Department of Endocrinology & Center of Clinical Investigation, Nantes University HospitalNantes, France; 4 Diabetes Centre, Royal Prince Alfred Hospital,University of SydneyCamperdown, Australia; 5 Núcleo Avançado de Netrologia, Hospital Sírio-LibanêsSão Paulo, Brazil; 6 Janssen Biotech, IncSpring House, PA, USA; 7 Janssen Research & Development, LLCRaritan, NJ, USA; 8 Janssen Research & DevelopmentBeerse, Belgium

**Keywords:** type 2 diabetes, sodium glucose co-transporter 2 (SGLT2) inhibitor, diabetic nephropathy

## Abstract

**Aims:**

Canagliflozin is a sodium glucose co-transporter 2 inhibitor in development for treatment of type 2 diabetes mellitus (T2DM). This study evaluated the efficacy and safety of canagliflozin in subjects with T2DM and stage 3 chronic kidney disease [CKD; estimated glomerular filtration rate (eGFR) ≥30 and <50 ml/min/1.73 m^2^].

**Methods:**

In this randomized, double-blind, placebo-controlled, phase 3 trial, subjects (N = 269) received canagliflozin 100 or 300 mg or placebo daily. The primary efficacy endpoint was change from baseline in HbA1c at week 26. Prespecified secondary endpoints were change in fasting plasma glucose (FPG) and proportion of subjects reaching HbA1c <7.0%. Safety was assessed based on adverse event (AE) reports; renal safety parameters (e.g. eGFR, blood urea nitrogen and albumin/creatinine ratio) were also evaluated.

**Results:**

Both canagliflozin 100 and 300 mg reduced HbA1c from baseline compared with placebo at week 26 (–0.33, –0.44 and –0.03%; p < 0.05). Numerical reductions in FPG and higher proportions of subjects reaching HbA1c < 7.0% were observed with canagliflozin 100 and 300 mg versus placebo (27.3, 32.6 and 17.2%). Overall AE rates were similar for canagliflozin 100 and 300 mg and placebo (78.9, 74.2 and 74.4%). Slightly higher rates of urinary tract infections and AEs related to osmotic diuresis and reduced intravascular volume were observed with canagliflozin 300 mg compared with other groups. Transient changes in renal function parameters that trended towards baseline over 26 weeks were observed with canagliflozin.

**Conclusion:**

Canagliflozin improved glycaemic control and was generally well tolerated in subjects with T2DM and Stage 3 CKD.

## Introduction

Progression of chronic kidney disease (CKD), leading to end-stage renal failure, is a common complication in patients with type 2 diabetes mellitus (T2DM) 1. Antihyperglycaemic agent (AHA) treatment options are limited as a number of classes of agents may have decreased efficacy and be associated with increased risk of adverse effects in patients with CKD 2. For example, there are labelled restrictions on the use of thiazolidinediones, metformin, sulphonylureas, and, more recently, glucagon-like peptide-1 (GLP-1) agonists in this cohort 3,4. Moreover, AHAs commonly used in this population, such as sulphonylureas, have been associated with an increased risk of hypoglycaemia and weight gain 3–5. Thus, new treatment options are needed for this growing population of patients with co-existing T2DM and renal insufficiency 1.

Canagliflozin is an inhibitor of the sodium glucose co-transporter 2 (SGLT2) in development for the treatment of patients with T2DM. Canagliflozin lowers the renal threshold for glucose (RT_G_) and increases urinary glucose excretion (UGE), resulting in decreased plasma glucose in patients with hyperglycaemia, as well as a mild osmotic diuresis and a net caloric loss (by loss of glucose) promoting weight loss 6–10. Because the rate of UGE is proportional to the glomerular filtration rate (GFR) (as well as to the blood glucose concentration) 7,8,11, the effect of canagliflozin to augment UGE would be anticipated to be diminished in subjects with CKD. Therefore, the efficacy of canagliflozin in improving glycaemic control and reducing body weight may be affected in this subject population. In addition to assessing the efficacy response to canagliflozin in this population, it is also important to assess the safety and tolerability profile of SGLT2 inhibition in subjects with CKD.

This phase 3 study evaluated the efficacy and safety of canagliflozin compared with placebo in subjects with inadequately controlled T2DM and stage 3 CKD. This study included subjects with a lower, more restricted estimated glomerular filtration rate (eGFR) range of ≥30 and <50 ml/min/1.73 m^2^ based upon the modification of diet in renal disease (MDRD) equation 12, compared with the typical eGFR range for stage 3 CKD of ≥30 and <60 ml/min/1.73 m^2^ [Bibr b13].

## Materials and Methods

### Study Design and Subjects

This 52-week, randomized, double-blind, placebo-controlled, phase 3 study was conducted at 89 centres in 19 countries and consisted of an AHA adjustment period (if required; consisting of a dose titration period of up to 4 weeks and an 8-week dose stable period); a 2-week, single-blind, placebo run-in period; a 26-week, double-blind, core treatment period; and a 26-week, double-blind, extension period (data to be reported in a separate publication). Eligible subjects were men and women aged ≥25 years with T2DM who had inadequate glycaemic control (HbA1c ≥7.0 and ≤10.5%) and stage 3 CKD (eGFR ≥30 and <50 ml/min/1.73 m^2^), and were either not on AHA therapy or were on a stable AHA regimen (monotherapy or combination therapy with any approved agent including metformin, sulphonylurea, dipeptidyl peptidase-4 (DPP-4) inhibitor, α-glucosidase inhibitor, GLP-1 analogue, pioglitazone or insulin) for ≥8 weeks (≥12 weeks with pioglitazone) prior to the week –2 visit. Subjects were required to have generally stable renal function, as determined by a ≤25% decrease in eGFR from the screening to the week –2 visits. Subjects on AHA regimens not consistent with local prescribing guidelines (e.g. metformin therapy) underwent an AHA adjustment period of up to 12 weeks before the placebo run-in period. Subjects were to remain on their stable AHA regimens through the completion of the 52-week treatment period (unless glycaemic rescue criteria were met, as discussed below).

Subjects were excluded if they had repeated fasting plasma glucose (FPG) >15.0 mmol/l (270 mg/dl) during the pretreatment phase; a history of T1DM; renal disease that required immunosuppressive therapy, dialysis or transplant; nephrotic syndrome or inflammatory renal disease; New York Heart Association Class III-IV cardiovascular disease; myocardial infarction, unstable angina, revascularization procedure or cerebrovascular accident within 3 months prior to screening; or haemoglobin concentration <100 g/l (10 g/dl) at screening.

The study protocol and amendments were approved by the institutional review boards at participating institutions and the study was conducted under the guidelines of Good Clinical Practices and the Declaration of Helsinki. All subjects provided written informed consent prior to participation.

### Randomization and Study Treatments

Eligible subjects were randomly assigned to receive once-daily oral doses of canagliflozin 100 or 300 mg or placebo in a 1 : 1 : 1 ratio using an Interactive Voice Response System/Interactive Web Response System. Randomization was balanced by using permuted blocks of six subjects per block and stratified based on (i) the presence or absence of atherosclerotic cardiovascular disease (e.g. history of myocardial infarction, documented angina, transient ischemic attack or stroke or peripheral vascular disease) and (ii) whether a subject required an AHA adjustment period prior to randomization.

During the double-blind, core treatment period, glycaemic rescue therapy (up-titration of current AHAs or step-wise addition of oral or non-oral AHAs) was initiated if FPG >15.0 mmol/l (270 mg/dl) after day 1 to week 6, >13.3 mmol/l (240 mg/dl) after week 6 to week 12, and >11.1 mmol/l (200 mg/dl) after week 12 to week 26. After randomization, HbA1c and FPG values were masked to the study centres unless these values met the prespecified glycaemic criteria for the initiation of rescue medication or after glycaemic rescue medication was started.

### Study Endpoints and Assessments

The prespecified primary efficacy endpoint was the change from baseline in HbA1c at week 26. Prespecified secondary efficacy endpoints evaluated at week 26 were the proportion of subjects reaching HbA1c <7.0% and change from baseline in FPG. Other efficacy endpoints included change from baseline in blood pressure (BP) and percent change from baseline in body weight and fasting plasma lipids.

Overall safety and tolerability were assessed by adverse event (AE) reports, safety laboratory tests, vital sign measurements, physical examinations and 12-lead electrocardiograms. Selected AEs of interest, including genital mycotic infections and urinary tract infections (UTIs), were prespecified for additional data collection. Events of hypoglycaemia were collected using a separate case report form that collected concurrent fingerstick glucose values and the presence of symptoms indicating a severe event (i.e. requiring the assistance of another individual or resulting in seizure or loss of consciousness). Measures of renal function, including eGFR, serum creatinine, blood urea nitrogen (BUN) and urine albumin/creatinine ratio (ACR) were also assessed.

### Statistical Analyses

Sample size calculation was based on demonstrating the superiority of canagliflozin to placebo, as measured by the change in HbA1c from baseline to week 26. An estimated 61 randomized subjects per group were needed to achieve ≥90% power, assuming a group difference of 0.5% and a common standard deviation (SD) of 0.85% (based on relevant information from patients with T2DM and renal impairment), and using a two-sample, two-sided *t*-test with a type I error rate of 0.05. In order to provide additional safety information for canagliflozin, this study planned a modestly greater sample size of 80 randomized subjects per treatment group (∼240 total subjects) for enrolment.

Efficacy analyses were conducted using the modified intent-to-treat (mITT) population, which consisted of all randomized subjects who received ≥1 dose of study drug, according to the randomized treatment assignment. The last observation carried forward (LOCF) approach was used to impute missing data. If subjects received rescue therapy, all postrescue data were censored and the last postbaseline value prior to the initiation of rescue therapy was used for analyses. Safety analyses were performed in randomized subjects who received ≥1 dose of study drug according to the predominant treatment received (the allocation of treatment assignment in the efficacy and safety analyses were the same as no subject took incorrect double-blind study drug for a predominant part of the double-blind treatment period).

Primary and continuous secondary efficacy endpoints were assessed using an analysis of covariance (ancova) model with treatment and stratification factors as fixed effects and corresponding baseline values and baseline eGFR as covariates. Least squares (LS) mean differences and two-sided 95% confidence intervals (CIs) were estimated based on this model for the comparison of each canagliflozin group versus placebo. The categorical secondary endpoint (proportion of subjects reaching HbA1c < 7.0%) was analyzed using a logistic model with treatment and stratification factors as fixed effects and baseline HbA1c and eGFR values as covariates. Renal safety parameters, including change in eGFR and ACR, were analyzed using an ancova model with treatment and stratification factors as fixed effects and adjusting for the baseline covariate. Differences in LS means between groups (each canagliflozin dose vs. placebo) and two-sided 95% CIs were estimated. All statistical tests were interpreted at a two-sided significance level of 5% and all CIs at a two-sided confidence level of 95%.

A closed testing of prespecified primary and secondary endpoints based on the treatment difference was implemented in order to preserve the overall type I error rate at 5%. The p values for the treatment comparisons were calculated and are reported for prespecified comparisons only. If a prespecified comparison was not found to be statistically significant, subsequent prespecified tests were not to be conducted; descriptive statistics (95% CI for between-group differences) are provided.

## Results

### Subject Disposition and Baseline Characteristics

**Figure 1:**
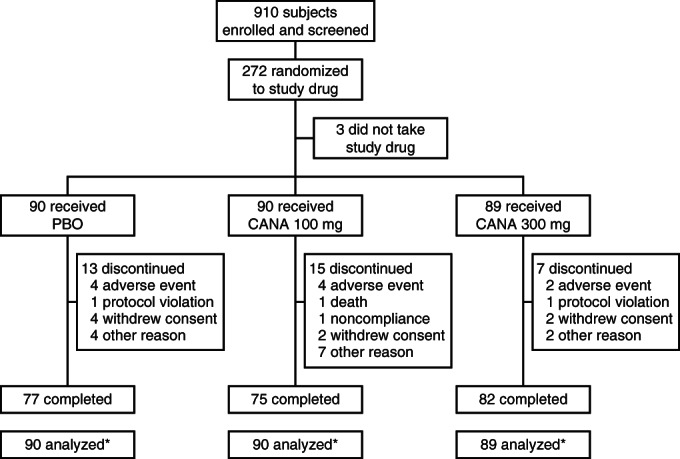
Study flow diagram. PBO, placebo; CANA, canagliflozin; mITT, modified intent-to-treat. *mITT analysis set.

Of the 272 randomized subjects, 269 received ≥1 dose of study drug and were included in the mITT analysis population (figure 1). A total of 35 (12.9%) subjects discontinued before the week 26 visit, with fewer discontinuations in the canagliflozin 300 mg group compared with the canagliflozin 100 mg and placebo groups. A smaller proportion of subjects treated with canagliflozin 100 or 300 mg received glycaemic rescue therapy before the week 26 visit compared with those treated with placebo (4.4, 3.3 and 14.3%, respectively). Baseline demographic and disease characteristics were similar across treatment groups (Table 1). Mean baseline HbA1c was 8.0%, mean age was 68.5 years and mean body mass index was 33.0 kg/m^2^; the mean duration of T2DM for subjects was 16.3 years. Mean baseline eGFR was 39.4 ml/min/1.73 m^2^ and median baseline ACR was 30.0 µg/mg. Approximately 80% of subjects had a history of ≥1 diabetic microvascular complication, with nephropathy being the most common complication. A total of 98% of subjects were on background AHA therapy at baseline, with insulin (74%) and sulphonylureas (31%) being the most common background therapies (Table 2). Most subjects were on antihypertensive therapy (Table 1), including agents acting on the renin-angiotensin system (87%), diuretics (73%), β-blocking agents (56%) and calcium channel blockers (42%).

**Table 1:** Baseline demographic and disease characteristics*

Characteristic	PBO (n = 90)	CANA 100 mg (n = 90)	CANA 300 mg (n = 89)	Total (N = 269)
Sex, n (%)				
Male	57 (63.3)	58 (64.4)	48 (53.9)	163 (60.6)
Female	33 (36.7)	32 (35.6)	41 (46.1)	106 (39.4)
Age, years	68.2 ± 8.4	69.5 ± 8.2	67.9 ± 8.2	68.5 ± 8.3
Race, n (%)†				
White	78 (86.7)	71 (78.9)	66 (74.2)	215 (79.9)
Black or African American	0	3 (3.3)	2 (2.2)	5 (1.9)
Asian	7 (7.8)	9 (10.0)	11 (12.4)	27 (10.0)
Other‡	5 (5.6)	7 (7.8)	10 (11.2)	22 (8.2)
HbA1c, %	8.0 ± 0.9	7.9 ± 0.9	8.0 ± 0.8	8.0 ± 0.9
FPG, mmol/l (mg/dl)	8.9 ± 2.4 (160.4 ± 43.2)	9.4 ± 2.6 (169.4 ± 46.3)	8.8 ± 3.2 (158.6 ± 58.0)	9.1 ± 2.8 (164.0 ± 49.6)
Body weight, kg	92.8 ± 17.4	90.5 ± 18.4	90.2 ± 18.1	91.2 ± 18.0
BMI, kg/m^2^	33.1 ± 6.5	32.4 ± 5.5	33.4 ± 6.5	33.0 ± 6.2
Duration of T2DM, years	16.4 ± 10.1	15.6 ± 7.4	17.0 ± 7.8	16.3 ± 8.5
eGFR, ml/min/1.73 m^2^	40.1 ± 6.8	39.7 ± 6.9	38.5 ± 6.9	39.4 ± 6.9
Median ACR, µg/mg	31.3	23.7	30.1	30.0
Microvascular complications, n (%)	74 (82.2)	73 (81.1)	69 (77.5)	216 (80.3)
Neuropathy	45 (50.0)	36 (40.0)	38 (42.7)	119 (44.2)
Retinopathy	25 (27.8)	27 (30.0)	36 (40.4)	88 (32.7)
Nephropathy	61 (67.8)	69 (76.7)	65 (73.0)	195 (72.5)
History of ASCVD, n (%)	51 (56.7)	50 (55.6)	46 (51.7)	147 (54.6)
Antihypertensive therapy at baseline, n (%)				
Agents acting on the renin-angiotensin system	77 (85.6)	79 (87.8)	79 (88.8)	235 (87.4)
Diuretics	62 (68.9)	65 (72.2)	70 (78.7)	197 (73.2)
β-blocking agents	50 (55.6)	51 (56.7)	50 (56.2)	151 (56.1)
Calcium channel blockers	33 (36.7)	40 (44.4)	39 (43.8)	112 (41.6)
Antihyperlipidemic therapy at baseline, n (%)	70 (77.8)	74 (82.2)	68 (76.4)	212 (78.8)

PBO, placebo; CANA, canagliflozin; FPG, fasting plasma glucose; BMI, body mass index; T2DM, type 2 diabetes mellitus; eGFR, estimated glomerular filtration rate; ACR, albumin/creatinine ratio; ASCVD, atherosclerotic cardiovascular disease; SD, standard deviation.

* Data are mean ± SD unless otherwise indicated.

† Percentages may not total 100.0% due to rounding.

‡ Includes American Indian or Alaska Native, Native Hawaiian or other Pacific Islander, other and unknown.

**Table 2:** AHA therapies at baseline (mITT)

	Subjects, n (%)			
	
	PBO (n = 90)	CANA 100 mg (n = 90)	CANA 300 mg (n = 89)	Total (N = 269)
Total subjects with AHA therapy	88 (97.8)	87 (96.7)	88 (98.9)	263 (97.8)
AHAs (alone or in combination)				
Sulphonylureas	33 (36.7)	24 (26.7)	27 (30.3)	84 (31.2)
Thiazolidinediones*	7 (7.8)	3 (3.3)	7 (7.9)	17 (6.3)
DPP-4 inhibitors	5 (5.6)	7 (7.8)	8 (9.0)	20 (7.4)
Biguanide	1 (1.1)	1 (1.1)	2 (2.2)	4 (1.5)
Other AHAs†	7 (7.8)	6 (6.7)	10 (11.2)	23 (8.6)
Insulin‡	66 (73.3)	67 (74.4)	66 (74.2)	199 (74.0)
Combinations§				
Sulphonylurea + insulin	11 (12.2)	7 (7.8)	10 (11.2)	28 (10.4)
Other AHA¶ + insulin	12 (13.3)	8 (8.9)	10 (11.2)	30 (11.2)
Biguanide + insulin	0	1 (1.1)	2 (2.2)	3 (1.1)
Biguanide + sulphonylurea	1 (1.1)	0	0	1 (0.4)

AHA, antihyperglycaemic agent; mITT, modified intent-to-treat; PBO, placebo; CANA, canagliflozin; DPP-4, dipeptidyl peptidase-4; GLP-1, glucagon-like peptide-1.

* All subjects were on pioglitazone.

† Including α-glucosidase inhibitors, GLP-1 agonists, glinides and other AHAs.

‡ Including basal + bolus insulin, basal insulin alone and bolus insulin alone.

§ Subset of subjects on combinations of the AHAs listed above.

¶ Including α-glucosidase inhibitors, thiazolidinediones, DPP-4 inhibitors, GLP-1 agonists, glinides and other AHAs.

### Efficacy

#### Glycaemic Efficacy Endpoints

**Figure 2:**
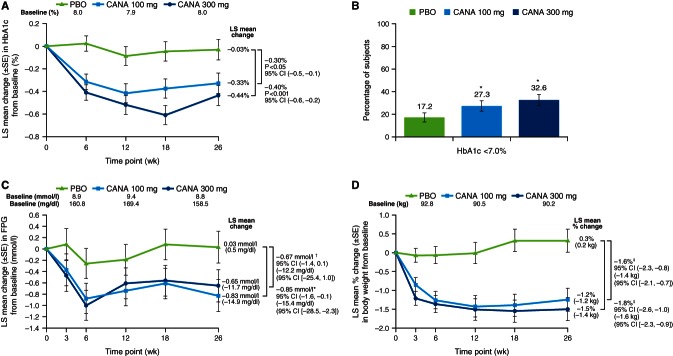
Effects on efficacy parameters (LOCF). Change in HbA1c (A), proportion of subjects reaching HbA1c <7.0% (B), change in FPG (C), and percent change in body weight (D). LOCF, last observation carried forward; FPG, fasting plasma glucose; PBO, placebo; CANA, canagliflozin; LS, least squares; SE, standard error; CI, confidence interval; NS, not significant. *Statistical comparison for CANA versus PBO not performed owing to multiplicity control. ^†^p = NS for CANA versus PBO. ^‡^Statistical comparison for CANA versus PBO not performed (not prespecified).

HbA1c was significantly reduced from baseline with canagliflozin 100 and 300 mg compared with placebo at week 26 (figure 2A). Differences in LS mean changes relative to placebo were –0.30% for canagliflozin 100 mg (p < 0.05) and –0.40% for canagliflozin 300 mg (p < 0.001). A numerically higher proportion of subjects treated with canagliflozin 100 or 300 mg than with placebo achieved HbA1c < 7.0% at week 26 (27.3, 32.6 and 17.2%, respectively; figure 2B). Canagliflozin 100 and 300 mg provided numerically greater reductions from baseline in FPG at week 26 compared with placebo (figure 2C), with differences in LS mean changes (95% CI) of –0.85 (–1.6, –0.1) and –0.67 (–1.4, 0.1) mmol/l [–15.4 (–28.5, –2.3) and –12.2 (–25.4, 1.0) mg/dl], respectively. The comparison of canagliflozin 300 mg versus placebo in change in FPG was not statistically significant and, therefore, the statistical comparison of canagliflozin 100 mg versus placebo did not proceed based on the closed testing procedure.

#### Other Efficacy Endpoints

Canagliflozin 100 and 300 mg provided reductions from baseline in body weight over 26 weeks, whereas placebo was associated with a slight increase in body weight (figure 2D). Differences in LS mean percent changes (95% CI) relative to placebo at week 26 were –1.6% (–2.3, –0.8) and –1.8% (–2.6, –1.0) for canagliflozin 100 and 300 mg, respectively, corresponding to absolute changes of –1.4 and –1.6 kg, respectively.

Both canagliflozin 100 and 300 mg were associated with greater decreases from baseline in systolic and diastolic BP compared with placebo at week 26 (LS mean changes of –6.1, –6.4 and –0.3 mmHg, respectively, in systolic BP and –2.6, –3.5 and –1.4 mmHg, respectively, in diastolic BP; Table 3). No notable changes in pulse rate were observed with canagliflozin 100 or 300 mg compared with placebo (mean change of –1.9, –1.1 and –2.5 beats/min, respectively). Canagliflozin 100 and 300 mg increased high-density lipoprotein cholesterol (HDL-C) compared with placebo (LS mean percent changes of 4.0, 3.0 and 1.5%, respectively; Table 3). An increase in triglycerides (LS mean percent changes of 11.9, 6.2 and 7.9%, respectively) and a decrease in low-density lipoprotein cholesterol (LDL-C; LS mean percent changes of –1.0, 6.4 and 6.3%, respectively) were seen with canagliflozin 300 mg compared with canagliflozin 100 mg and placebo. There was no notable difference in non–HDL-C between the canagliflozin 300 mg and placebo groups (LS mean percent changes of 2.8 and 3.8%, respectively).

**Table 3:** Summary of blood pressure and fasting plasma lipid findings at week 26 (LOCF)*

Parameter	PBO (n = 90)	CANA 100 mg (n = 90)	CANA 300 mg (n = 89)
Systolic BP, n	89	90	89
Mean ± SD baseline, mmHg	132.1 ± 13.6	135.9 ± 13.1	136.7 ± 15.0
LS mean ± SE change	–0.3 ± 1.5	–6.1 ± 1.5	–6.4 ± 1.5
Difference versus PBO (95% CI)		–5.7 (–9.5, –1.9)	–6.1 (–10.0, –2.3)
Diastolic BP, n	89	90	89
Mean ± SD baseline, mmHg	73.9 ± 9.0	73.5 ± 8.8	75.7 ± 7.8
LS mean ± SE change	–1.4 ± 0.9	–2.6 ± 0.9	–3.5 ± 0.9
Difference versus PBO (95% CI)		–1.2 (–3.4, 1.0)	–2.1 (–4.3, 0.2)
Triglycerides, n	75	82	85
Mean ± SD baseline, mmol/l (mg/dl)	2.0 ± 1.1 (179.4 ± 96.2)	1.9 ± 0.9 (164.9 ± 81.1)	2.1 ± 1.2 (189.3 ±103.5)
LS mean ± SE change, mmol/l (mg/dl)	–0.01 ± 0.11 (–0.7 ± 10.0)	0.02 ± 0.11 (1.8 ± 9.7)	0.22 ± 0.11 (19.5 ± 9.5)
Median (IQR) percent change	2.8 (–18.4, 22.8)	–1.0 (–20.3, 17.3)	2.5 (–16.1, 25.8)
LS mean ± SE percent change	7.9 ± 4.8	6.2 ± 4.6	11.9 ± 4.6
Difference versus PBO (95% CI)		–1.7 (–13.8, 10.5)	3.9 (–8.1, 15.9)
LDL-C, n	75	82	84
Mean ± SD baseline, mmol/l (mg/dl)	2.5 ± 1.0 (96.3 ± 39.0)	2.4 ± 0.9 (91.3 ± 33.4)	2.3 ± 0.9 (87.2 ± 33.9)
LS mean ± SE change, mmol/l (mg/dl)	0.06 ± 0.08 (2.4 ± 3.0)	0.09 ± 0.08 (3.5 ± 2.9)	–0.08 ± 0.08 (–3.1 ± 2.9)
Median (IQR) percent change	0.0 (–14.2, 22.3)	1.3 (–9.0, 15.5)	0.2 (–16.7, 16.1)
LS mean ± SE percent change	6.3 ± 3.6	6.4 ± 3.5	–1.0 ± 3.4
Difference versus PBO (95% CI)		0.1 (–8.9, 9.2)	–7.2 (–16.3, 1.8)
HDL-C, n	75	82	85
Mean ± SD baseline, mmol/l (mg/dl)	1.1 ± 0.3 (42.6 ± 10.0)	1.1 ± 0.2 (43.1 ± 8.6)	1.2 ± 0.3 (44.3 ± 12.2)
LS mean ± SE change, mmol/l (mg/dl)	0.00 ± 0.02 (0.1 ± 0.7)	0.03 ± 0.02 (1.3 ± 0.7)	0.02 ± 0.02 (0.8 ± 0.7)
Median (IQR) percent change	1.6 (–7.3, 8.2)	2.3 (–6.6, 10.9)	2.1 (–7.3, 11.8)
LS mean ± SE percent change	1.5 ± 1.8	4.0 ± 1.7	3.0 ± 1.7
Difference versus PBO (95% CI)		2.5 (–1.9, 7.0)	1.5 (–3.0, 5.9)
LDL-C/HDL-C, n	75	82	84
Mean ± SD baseline, mol/mol	2.3 ± 1.0	2.2 ± 0.9	2.1 ± 0.8
LS mean ± SE change	0.04 ± 0.08	0.02 ± 0.07	–0.15 ± 0.07
Median (IQR) percent change	–0.5 (–12.9, 16.6)	–2.2 (–13.0, 13.8)	3.3 (–19.5, 12.4)
LS mean ± SE percent change	4.7 ± 3.8	4.7 ± 3.7	–4.3 ± 3.7
Difference versus PBO (95% CI)		0.0 (–9.7, 9.7)	–8.9 (–18.6, 0.8)
Non–HDL-C, n	75	81	85
Mean ± SD baseline, mmol/l (mg/dl)	3.4 ± 1.1 (131.9 ± 41.9)	3.2 ± 0.9 (123.9 ± 36.2)	3.3 ± 1.0 (125.7 ± 39.4)
LS mean ± SE change, mmol/l (mg/dl)	0.06 ± 0.09 (2.2 ± 3.5)	0.10 ± 0.09 (4.0 ± 3.4)	0.02 ± 0.09 (0.9 ± 3.4)
Median (IQR) percent change	–1.0 (–13.7, 15.0)	0.8 (–9.3, 12.0)	1.9 (–11.5, 12.0)
LS mean ± SE percent change	3.8 ± 2.9	5.1 ± 2.8	2.8 ± 2.8
Difference versus PBO (95% CI)		1.2 (–6.1, 8.6)	–1.1 (–8.3, 6.2)

LOCF, last observation carried forward; PBO, placebo; CANA, canagliflozin; BP, blood pressure; SD, standard deviation; LS, least squares; SE, standard error; CI, confidence interval; IQR, interquartile range; LDL-C, low-density lipoprotein cholesterol; HDL-C, high-density lipoprotein cholesterol.

* Statistical comparison for CANA 100 and 300 mg versus PBO not performed (not prespecified).

### Safety

#### Overall Safety and Tolerability

The overall incidence of AEs, serious AEs and study discontinuations due to AEs was similar for canagliflozin 100 and 300 mg and placebo (Table 4). The incidence of drug-related AEs was higher in both canagliflozin groups compared with placebo, largely because of a higher incidence of several specific AEs discussed below.

**Table 4:** Summary of overall safety and selected AEs*

	Subjects, n (%)		
	
	PBO (n = 90)	CANA 100 mg (n = 90)	CANA 300 mg (n = 89)
Any AE	67 (74.4)	71 (78.9)	66 (74.2)
AEs leading to discontinuation	5 (5.6)	4 (4.4)	2 (2.2)
AEs related to study drug†	20 (22.2)	23 (25.6)	29 (32.6)
Serious AEs	16 (17.8)	10 (11.1)	10 (11.2)
Deaths	1 (1.1)	1 (1.1)	0
Selected AEs			
UTI	5 (5.6)	5 (5.6)	7 (7.9)
Genital mycotic infection			
Male†,§	0	1 (1.7)	1 (2.1)
Female¶,‖	0	1 (3.1)	1 (2.4)
Osmotic diuresis-related AEs			
Pollakiuria**	1 (1.1)	2 (2.2)	4 (4.5)
Polyuria††	0	0	0
Volume-related AEs			
Postural dizziness	0	1 (1.1)	2 (2.2)
Orthostatic hypotension	0	0	1 (1.1)

AE, adverse event; PBO, placebo; CANA, canagliflozin; UTI, urinary tract infection.

* All AEs are reported for regardless of rescue medication, except for osmotic diuresis- and volume-related AEs, which are reported for prior to initiation of rescue therapy.

† Possibly, probably or very likely related to study drug, as assessed by investigators.

‡ PBO, n = 57; CANA 100 mg, n = 58; CANA 300 mg, n = 48.

§ Including balanitis and posthitis.

¶ PBO, n = 33; CANA 100 mg, n = 32; CANA 300 mg, n = 41.

‖ Including vulvovaginal mycotic infection.

** Increased urine frequency.

†† Increased urine volume.

Canagliflozin was associated with slightly higher rates of genital mycotic infections in males and females compared with placebo (Table 4), but incidences were low across groups and none led to study discontinuation. The incidence of UTIs was higher with canagliflozin 300 mg compared with canagliflozin 100 mg and placebo, with no upper UTI AEs reported. All events were considered by investigators to be mild or moderate in severity, with none leading to study discontinuation. Incidences of pollakiuria (increased urine frequency) and AEs related to reduced intravascular volume (i.e. postural dizziness and orthostatic hypotension) were increased with canagliflozin 300 mg relative to canagliflozin 100 mg and placebo; these were low across groups, generally mild or moderate in intensity and infrequently led to discontinuation. There was no report of polyuria (increased urine volume) in any group.

Most subjects (96.3%) were on background AHA therapy associated with an increased risk of hypoglycaemia (i.e. insulin or sulphonylurea agents). Among these subjects, the proportion with documented hypoglycaemia episodes was higher with canagliflozin 100 and 300 mg (52.9 and 51.2%, respectively) compared with placebo (36.4%). Six subjects experienced severe hypoglycaemia episodes [4 (4.7%), 1 (1.2%) and 1 (1.1%) with canagliflozin 100 and 300 mg and placebo, respectively]. There were no documented hypoglycaemia episodes reported among subjects who were not on insulin or a sulphonylurea agent.

Overall, only small differences in safety laboratory parameters were observed with canagliflozin 100 and 300 mg relative to placebo (Table 5). At week 26, similar increases in alanine aminotransferase (ALT) and aspartate aminotransferase (AST) were observed with canagliflozin 100 mg (mean percent changes of 10.1 and 5.5%, respectively) and placebo (8.2 and 4.3%, respectively), whereas decreases were seen with canagliflozin 300 mg (–4.4 and –4.3%, respectively). Increases in serum magnesium were seen with canagliflozin 100 and 300 mg, whereas no change was observed with placebo (mean percent changes of 9.1, 14.6 and 0.0%, respectively). Dose-related increases in serum phosphate were seen with canagliflozin 100 and 300 mg compared with placebo (mean percent changes of 4.9, 9.5 and 1.0%, respectively). Canagliflozin 100 and 300 mg were associated with non–dose-related increases in haemoglobin compared with a minimal change with placebo (mean percent changes of 5.3, 3.1 and –0.5%, respectively); corresponding changes in haematocrit were observed (mean percent changes of 6.0, 4.8 and –0.1%, respectively; Table 5).

**Table 5:** Mean percent changes in clinical laboratory parameters from baseline to week 26*

	PBO	CANA 100 mg	CANA 300 mg
ALT, n	63	70	78
Mean baseline, U/l	23.7	20.8	22.9
Mean ± SD percent change	8.2 ± 48.5	10.1 ± 40.4	–4.4 ± 34.8
Alkaline phosphatase, n	63	70	78
Mean baseline, U/l	79.3	77.8	80.2
Mean ± SD percent change	5.3 ± 17.7	7.0 ± 19.6	–2.1 ± 15.5
AST, n	62	67	78
Mean baseline, U/l	23.6	21.9	23.7
Mean ± SD percent change	4.3 ± 30.9	5.5 ± 31.3	–4.3 ± 20.7
Bilirubin, n	63	70	78
Mean baseline, µmol/l	7.7	8.2	8.1
Mean ± SD percent change	4.1 ± 31.6	4.5 ± 31.9	7.4 ± 41.8
Magnesium, n	63	70	78
Mean baseline, mmol/l	0.8	0.8	0.8
Mean ± SD percent change	0.0 ± 9.3	9.1 ± 10.4	14.6 ± 12.9
Phosphate, n	63	70	77
Mean baseline, mmol/l	1.2	1.2	1.2
Mean ± SD percent change	1.0 ± 16.5	4.9 ± 16.0	9.5 ± 20.5
Urate, n	63	70	78
Mean baseline, µmol/l	433.7	434.4	442.5
Mean ± SD percent change	2.5 ± 18.6	–0.3 ± 16.9	–2.0 ± 20.0
Haemoglobin, n	62	69	76
Mean baseline, g/l	136.2	133.8	130.9
Mean ± SD percent change	–0.5 ± 8.1	5.3 ± 7.4	3.1 ± 5.9
Haematocrit, n	62	69	76
Mean baseline, %	40.8	40.1	39.2
Mean ± SD percent change	–0.1 ± 9.1	6.0 ± 7.6	4.8 ± 6.9

PBO, placebo; CANA, canagliflozin; ALT, alanine aminotransferase; SD, standard deviation; AST, aspartate aminotransferase.

* Statistical comparison for CANA 100 and 300 mg versus PBO not performed (not prespecified).

##### Measures of Renal Function

**Figure 3:**
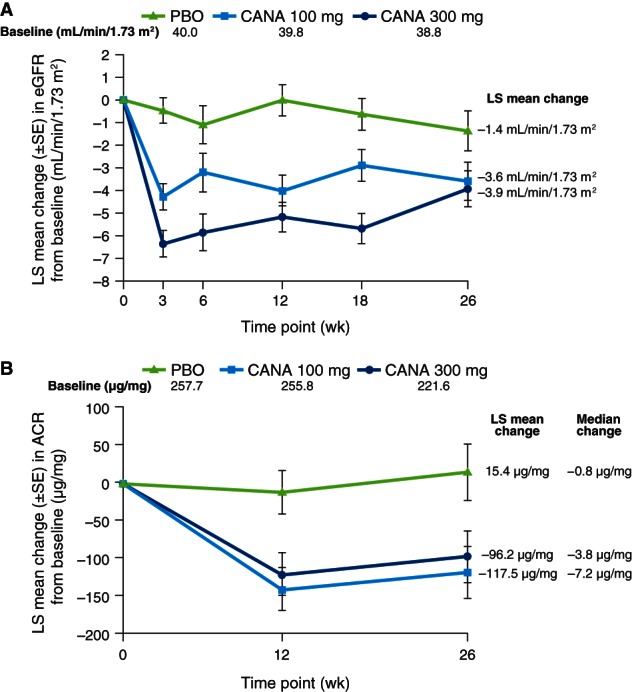
Change in eGFR (A) and ACR (B) over time. ^#^eGFR, estimated glomerular filtration rate; ACR, albumin/creatinine ratio; PBO, placebo; CANA, canagliflozin; LS, least squares; SE, standard error. *Statistical comparison for CANA versus PBO not performed (not prespecified).

Changes in renal function parameters were observed with both canagliflozin doses compared with placebo. Decreases in eGFR from baseline were observed in all treatment groups and were larger in the canagliflozin 100 and 300 mg groups relative to the placebo group: LS mean percent changes of –9.1, –10.1 and –4.5%, respectively. The reductions in eGFR with canagliflozin were largest at week 3 (the first postbaseline measurement) and then trended back towards baseline over the 26-week treatment period (figure 3A). Increases in BUN were observed with canagliflozin 100 and 300 mg compared with placebo (LS mean percent changes of 12.1, 12.5 and 4.9%, respectively); these increases also occurred early and then trended towards baseline over the remaining treatment period. Canagliflozin 100 and 300 mg were associated with greater decreases in urine ACR compared with placebo, with median percent reductions of –29.9, –20.9 and –7.5%, respectively (figure 3B). Progression of albuminuria from baseline to week 26 was examined (i.e. from normoalbuminuria to micro- or macroalbuminuria, or from micro- to macroalbuminuria), with a lower proportion of subjects in the canagliflozin 100 and 300 mg groups progressing relative to those in the placebo group [5.1, 8.3 and 11.8%, respectively; odds ratio (95% CI) of 0.33 (0.08, 1.48) and 0.51 (0.14, 1.91) for the pairwise comparisons of canagliflozin 100 and 300 mg to placebo, respectively].

## Discussion

This study examined the efficacy and safety of canagliflozin in subjects with T2DM and stage 3 CKD, but in a lower, more restricted eGFR range of this classification (i.e. 30 to <50 ml/min/1.73 m^2^ rather than to <60 ml/min/1.73 m^2^). In this study population, both canagliflozin doses significantly lowered HbA1c compared with placebo over 26 weeks of therapy. Reductions in HbA1c with canagliflozin 100 and 300 mg (differences in LS mean changes vs. placebo of –0.30 and –0.40%, respectively) were clinically useful, particularly in the setting of stage 3 CKD in which available oral AHA therapy options are limited.

In addition to providing reductions in HbA1c, a numerically greater proportion of subjects treated with canagliflozin (including about a third of subjects in the canagliflozin 300 mg group) achieved HbA1c <7.0% than those treated with placebo, indicating that canagliflozin provides meaningful clinical value in this patient population. Both canagliflozin doses provided numerically greater reductions in FPG compared with placebo, although these differences did not achieve statistical significance. A smaller proportion of subjects treated with canagliflozin required glycaemic rescue therapy compared with placebo-treated subjects. Both canagliflozin doses were also associated with reductions in body weight relative to placebo. The mechanism by which canagliflozin reduces body weight is thought to be related to the loss of calories associated with induction of UGE, although through the osmotic diuretic effect, reduced fluid volume may also contribute to the reduction in body weight – this may be particularly relevant in patients with renal impairment who tend to have sodium and fluid retention, and therefore have excessive fluid volume. Also likely related to the osmotic diuretic effect, canagliflozin provided numerically greater reductions in systolic and diastolic BP compared with placebo, an important observation given the often difficult-to-treat hypertension in patients with stage 3 CKD.

The efficacy on glycaemic parameters and body weight reduction observed with canagliflozin in this population of subjects was less than that seen in subjects with T2DM who have normal or only mildly impaired renal function 10,14–17. This is not unexpected because the rate of UGE is related to both plasma glucose concentration and eGFR; with lower eGFR, the ability of canagliflozin to augment UGE is attenuated 7,8,11. With lesser increases in UGE, the glucose-lowering efficacy of canagliflozin is also reduced. Results from the current study are consistent with those from a phase 1 canagliflozin study showing reduced UGE and decreased RT_G_ lowering in subjects with stage 3 CKD compared with subjects with normal renal function 18. Owing to the limited amount of UGE observed with canagliflozin treatment in patients with more severe renal insufficiency 18, SGLT2 inhibitors are not expected to be efficacious for patients with an eGFR <30 ml/min/1.73 m^2^ (i.e. stages 4 or 5 CKD) or for dialysis patients.

Both canagliflozin doses were well tolerated, and incidences of AEs, serious AEs and study discontinuations due to AEs were similar across treatment groups. The AEs associated with SGLT2 inhibition seen in other canagliflozin phase 3 studies 14–17, including genital mycotic infections, a small increase in UTIs (with no reports of upper UTI AEs), and AEs related to osmotic diuresis (i.e. pollakiuria and polyuria), were also seen in this study, although at lower rates, which may reflect the attenuation of UGE in this study population.

Canagliflozin acts by lowering RT_G_; this value is typically 10.0 mmol/l (180 mg/dl) in normal individuals, raised in patients with T2DM to approximately 13.3 mmol/l (240 mg/dl), and reduced to levels of approximately 4.4 to 5.0 mmol/l (80-90 mg/dl) in patients treated with canagliflozin 9,10. Because the usual threshold for hypoglycaemia is approximately 3.9 mmol/l (70 mg/dl), this would suggest a low risk for hypoglycaemia with canagliflozin – as has been observed in studies of healthy volunteers and patients with T2DM 7,9,10,16. When an agent not associated with hypoglycaemia is added to the regimen of a medication that is associated with hypoglycaemia, like insulin or a sulphonylurea agent, an increase in hypoglycaemia is usually observed 19–23. This was seen in this study for subjects on background therapy with insulin or a sulphonylurea agent, in whom both canagliflozin doses were associated with the expected higher incidences of hypoglycaemia relative to placebo. Importantly, the rate of severe hypoglycaemia in subjects on such agents was low and there were no documented hypoglycaemia episodes among subjects not on insulin or a sulphonylurea agent.

Because the kidney is a target organ with canagliflozin treatment, the effects of canagliflozin on renal function were carefully assessed in this study. Canagliflozin 100 and 300 mg were associated with some changes in renal function (assessed by eGFR, serum creatinine, BUN and ACR) early on, with subsequently stable or improving eGFR values over the 26-week core treatment period (compared with a gradual, small decline in the placebo group). These transient changes in renal function with canagliflozin may be related to a mild osmotic diuretic effect of this agent, with small reductions in plasma volume, leading to a mild prerenal pattern. The proportion of subjects with progression of albuminuria with canagliflozin was slightly less than with placebo; the decrease in the ACR along with the stable renal function after the small initial decline is reassuring with regard to the lack of renal injury with this agent. The urinary ACR has been used as a biomarker, with reduction suggesting prevention of progression of renal injury 24,25, as seen with angiotensin-converting enzyme (ACE) inhibitors or angiotensin receptor blockers (ARBs); whether the reduction in ACR seen with canagliflozin, along with stable renal function (after the small initial decrease), indicates the potential for renal protection with canagliflozin can only be assessed with longer-term and larger studies.

As noted, the efficacy observed with canagliflozin in this renal-impaired population with T2DM was less than that observed in subjects with normal or only mildly impaired renal function. Nonetheless, this agent still provides important clinical value in this setting. It is important to note that physicians managing such patients have limited options, with several agents restricted (e.g. metformin or thiazolidinediones), and other agents that must be used carefully owing to safety concerns, including sulphonylurea agents and insulin that can lead to sodium retention, weight gain and hypoglycaemia. Treatment with canagliflozin added on to subjects' stable diabetes treatment regimens lowered HbA1c and resulted in more patients reaching HbA1c goal compared with placebo, indicating clinical utility. Additional studies are needed to assess the efficacy and safety of canagliflozin monotherapy in patients with renal impairment. It is interesting to note that another SGLT2 inhibitor, dapagliflozin, has not demonstrated HbA1c-lowering efficacy in this patient population 26; whether this indicates differences between agents in this class or differences in study design remains to be determined.

In conclusion, canagliflozin 100 and 300 mg significantly reduced HbA1c and were associated with numerical reductions in FPG, body weight and BP compared with placebo after 26 weeks of therapy in subjects with T2DM and stage 3 CKD (eGFR ≥30 and <50 ml/min/1.73 m^2^). Canagliflozin was generally well tolerated, with an expected increase in hypoglycaemia among the >95% of subjects on insulin or a sulphonylurea agent. Canagliflozin was associated with transient changes in renal function parameters that recovered towards baseline over the study period. These findings suggest that canagliflozin may be an appropriate treatment option for patients with T2DM and stage 3 CKD.
